# Novel Plasmids and Resistance Phenotypes in *Yersinia pestis*: Unique Plasmid Inventory of Strain Java 9 Mediates High Levels of Arsenic Resistance

**DOI:** 10.1371/journal.pone.0032911

**Published:** 2012-03-30

**Authors:** Mark Eppinger, Lyndsay Radnedge, Gary Andersen, Nicholas Vietri, Grant Severson, Sherry Mou, Jacques Ravel, Patricia L. Worsham

**Affiliations:** 1 Institute for Genome Sciences and Department of Microbiology and Immunology, University of Maryland, School of Medicine, Baltimore, Maryland, United States of America; 2 Lawrence Livermore National Laboratory, Livermore, California, United States of America; 3 United States Army Medical Research Institute of Infectious Diseases, Bacteriology Division, Fort Detrick, Maryland, United States of America; University of Minnesota, United States of America

## Abstract

Growing evidence suggests that the plasmid repertoire of *Yersinia pestis* is not restricted to the three classical virulence plasmids. The Java 9 strain of *Y. pestis* is a biovar Orientalis isolate obtained from a rat in Indonesia. Although it lacks the *Y. pestis*-specific plasmid pMT, which encodes the F1 capsule, it retains virulence in mouse and non-human primate animal models. While comparing diverse *Y. pestis* strains using subtractive hybridization, we identified sequences in Java 9 that were homologous to a *Y. enterocolitica* strain carrying the transposon *Tn2502*, which is known to encode arsenic resistance. Here we demonstrate that Java 9 exhibits high levels of arsenic and arsenite resistance mediated by a novel promiscuous class II transposon, named *Tn2503*. Arsenic resistance was self-transmissible from Java 9 to other *Y. pestis* strains via conjugation. Genomic analysis of the atypical plasmid inventory of Java 9 identified pCD and pPCP plasmids of atypical size and two previously uncharacterized cryptic plasmids. Unlike the *Tn2502*-mediated arsenic resistance encoded on the *Y. enterocolitica* virulence plasmid; the resistance loci in Java 9 are found on all four indigenous plasmids, including the two novel cryptic plasmids. This unique mobilome introduces more than 105 genes into the species gene pool. The majority of these are encoded by the two entirely novel self-transmissible plasmids, which show partial homology and synteny to other enterics. In contrast to the reductive evolution in *Y. pestis*, this study underlines the major impact of a dynamic mobilome and lateral acquisition in the genome evolution of the plague bacterium.

## Introduction

*Yersinia pestis*, the causative agent of plague, is a clonal descendant of *Y. pseudotuberculosis* serotype O:1b and is thought to have originated in modern day China [1], [2]. The divergence of the two species was estimated to have occurred within the last 20,000 years [3], resulting in a genetically highly homogenous population structure [4], [5]. The epidemiology of plague in Indonesia has been studied in numerous field surveys [6], [7], [8], [9], [10], [11], [12]. The atypical Indonesian strain described here, Java 9, lacks the *Y. pestis*-specific plasmid pMT, which encodes the antiphagocytic F1 capsule. However, this strain is fully virulent in rodent and non-human primate models [13]. Genotypic profiling of strain Java 9 based on the tetranucleotide (CAAA)_n_ repeat sequence or VNTR (variable number of tandem repeats) [14] and a set of difference regions (DFR) [15] suggests its phylogenetic assignment to the *Orientalis* branch (1.ORI1), which is further supported by the SNP-derived phylogenetic analysis of related isolates from Java [3], [5]. Phenotypically, Java 9 does not ferment glycerol, rhamnose, or melibiose and reduces nitrate (66). The pCD and pPCP plasmids of this strain are of atypical size and a cryptic plasmid was previously reported (60).

Cryptic plasmids have been described in the literature as part of the *Y. pestis* mobilome [16], but in many cases, no sequence data are available to decipher the nature and impact of such plasmids on epidemiology and the pathogenesis of the organism [17]. Unusual sizes among the typical virulence plasmids were previously attributed to intrachromosomal deletions, lateral acquisition of genomic fragments, and chimerical plasmid architectures [1], [4], [18].

Chromosomal and plasmid-mediated arsenic resistance has been described in a number of gram-negative and gram-positive bacteria and archaea. In response to arsenic toxicity, microbes have evolved mechanisms for arsenic resistance involving enzymes that oxidize As(III) to As(V) or reduce As(V) to As(III) [19]. Toxic metal resistance operons are induced by the arsenic resistance regulator ArsR and mediated by the expulsion of arsenite anions by a specific anion pump (ArsB), while arsenate is first converted to arsenite by an arsenate reductase (ArsC) before extrusion [19], [20]. Another protein specifically required for arsenic resistance in *Y. enterocolitica* [21] is the NADPH-dependent FMN reductase domain containing protein ArsH, while its role in other species remains unclear [22], [23]. In serotype O3 strains of *Y. enterocolitica*, arsenic resistance has been associated with a copy of the transposon *Tn2502* carried on pYV, the *Yersinia* virulence plasmid responsible for the low calcium response. [21]. The *arsHRBC* operon encoded by *Tn2502* has a high degree of similarity to the chromosomal *arsRBC* operon of *Escherichia coli* [24]. Arsenic resistance in the genus *Yersinia* is not limited to *Y. enterocolitica*; it has also been reported in a panel of *Y. intermedia* and other *Y. enterocolitica* serovars. However, the underlying genetic mechanisms in these organisms have not been determined [25]. This study describes the phenotypic and genotypic characterization of arsenic resistance in *Y. pestis* Java 9. Genomic analysis of the unique mobilome of strain Java 9 revealed the molecular nature of the conjugal transfer and detoxification loci that mediate its unique resistance to arsenic and transmissibility of the resistance phenotype.

## Materials and Methods

### Ethics statement. “N/A”. Ethics committee waived the need for consent

#### Bacterial strains

*Y. pestis* Java 9 is a fully virulent pMT(-) deficient biovar ORI strain isolated in 1957 from a dead Javanese rat (*Rattus rattus diardi*) in the village of Gempolan, Java, Indonesia. The original glycerol stock dating back to 1957 has been cultivated only once for single colony DNA isolation and sequencing. Material for sequencing was taken from a master seed stock prepared after mouse passage to investigate its virulence phenotype. There was one passage between the frozen vial and the mouse derived isolate. A global collection of *Y. pestis* strains was screened for arsenic resistance (ars) and prevalence of transposon *Tn2503* and conjugal transfer plasmids, and further used in conjugation experiments (**Table S2**).

#### Screening for Java 9-specific loci

PCR reactions were performed with 1 unit of *Taq* polymerase (Roche) in the supplied buffer. PCR amplification reaction mixtures contained 10 µM of each primer and 1 mM dNTPs. The PCR program involved one step at 94°C for 5 min, followed by 35 cycles of amplification of three steps (i) 94°C for 30 s, (ii) 60°C for 30 s and (iii) 72°C for 7 min. PCR products were maintained at 72°C for 7 min, separated by gel electrophoresis in 1% agarose gels, and stained with ethidium bromide (EtBr). The primer pairs are listed in **Table S3.**

#### Growth Media

Unless otherwise indicated, 1.25 mM sodium arsenate was added to trypticase soy agar (TSA) or Congo red agar for selection and screening of arsenic-resistant strains. When used, ampicillin was present at 30 µicrog/ml. Glycerol fermentation was evaluated using 0.2% glycerol as the sole fermentable carbon source with bromothymol blue as pH indicator. Tryptose blood agar (TBA) base (Difco) containing magnesium oxalate (20 mM) and MgCl_2_ (20 mM) was employed to cure pCD. We used heart infusion broth (HIB, Difco) containing 0.2% xylose as a general-purpose liquid culture medium.

#### Mating Experiments

Plate and broth matings were performed as described by Worsham et al to select for arsenic sensitivity and resistance [26]. Media for plate matings were Amp/As TSA agar or Congo red/As agar; broth matings took place in HIB/xylose with subsequent plating on selective media. Where possible, recipient strains were chosen with a marker useful for counter selection (Amp^r^) against the donor. In other cases, a differential medium (Congo red agar) was used to screen mating mixes to phenotypically identify the recipient strain. The genetic background of the putative transconjugants was confirmed by PCR (*caf1, lcrV, pla*) and/or biochemical analysis (glycerol fermentation).

#### Plasmid sequencing and annotation

Plasmid DNA of *Y. pestis* Java 9 (Project ID: 49905) was subjected to random shotgun sequencing and closure strategies as previously described [4]. Random insert libraries of 3 to 5 kb and 10 to 12 kb were constructed. A draft genome sequence was assembled using the Celera assembler [27]. An estimate of the copy number ratios of each plasmid was obtained by dividing the coverage depth of the plasmid. The four plasmids were manually annotated using the IGS Manatee system (http://manatee.sourceforge.net/).

#### Plasmid visualization and comparisons

The Chi-squares and GC-skews were computed according to methods described in [4]. For the Chi-square, a window size of 1 kb and a sliding window of 0.2 kb were used for the four plasmids, GC-skews were calculated using a window size of 0.2 kb. The whole genome alignment tool NUCmer [28] was used to calculate the overall gene identities among the analyzed plasmid molecules. For each of the predicted plasmid proteins of *Y. pestis* Java 9, a BLASTP raw score was obtained for the alignment against itself (REF_SCORE) and the most similar protein (QUE_SCORE). These scores were normalized by dividing the QUE_SCORE obtained for each query genome protein by the REF_SCORE. Proteins with a normalized ratio of <0.4 were considered to be non-homologous. A normalized BLAST score ratio of 0.4 is generally similar to two proteins being 30% identical over their entire length [29].

#### Plasmid copy numbers

To determine the plasmid copy numbers, the insertion of the 6,769 bp transposon *Tn2503* was excised *in silico* in the individual plasmid sequences. Copy numbers for each plasmid assembly were estimated based on the level of sequence read coverage with a nucleotide level identity threshold greater than 99%.

#### Transposon polymorphisms

Single nucleotide polymorphisms (SNP) were identified by comparing transposon *Tn2503* to *Tn2502* using a bioinformatics SNP discovery and validation pipeline [4], [30]. By mapping the position of each SNP to the *Tn2503* annotation, it was possible to determine the effect on the deduced polypeptide and classify each SNP as synonymous or non-synonymous.

#### Nucleotide Sequence Accession Numbers

The plasmid sequences have been deposited in GenBank under accession numbers CP002179 (pJARS35), CP002180p (pPCP), CP002181 (pJARS36) and CP002182 (pCD).

## Results

### *Y. pestis* strain Java 9 is resistant to arsenic

While comparing the genomes of diverse *Y. pestis* strains using subtractive hybridization, we identified DNA sequences in Java 9 that were homologous to *Tn2502*, a transposon found in certain serotypes of *Y. enterocolitica* [21]. We screened Java 9 and a set of genetically diverse *Y. pestis* strains (**Table S2**) for resistance to arsenate and arsenite. Java 9 was resistant to >1.25 mM sodium m-arsenite and >100 mM sodium arsenate. Significant inhibition was observed with 10 mM arsenite. This was at least 32-fold higher than the arsenite resistance of the other 10 *Y. pestis* strains tested, including CO92, La Paz, 1171, KIM10, 195P-3, Antigua, Nairobi, Pestoides A, EV76, and A1122. We tested also another panel of six Javanese strains that are in the USAMRID collection that were sensitive to arsenic compounds (data not shown).When tested on TBA plates containing 2.5 mM CaCl_2_ (to allow growth of pYV^+^ strains at 37°C) and a range of sodium arsenate concentrations, arsenic resistance appeared to be expressed equally well at both 28°C and 37°C.

### Arsenic resistance does not require pCD (pYV)

Because arsenic resistance in some strains of *Y. enterocolitica* is encoded by pYV, the *Yersinia* virulence plasmid that mediates the low calcium response, we cured Java 9 of pCD, as the plasmid is known in *Y. pestis*, by culturing at 37°C on a growth medium containing magnesium oxalate. Plasmid profile analysis indicated that the resulting strains lacked pCD and PCR analysis confirmed the loss of the pCD -encoded gene *lcrV*. The resulting strain retained its resistance to arsenic and arsenite. Thus, resistance to arsenic in Java 9 does not require pCD-encoded sequences.

### Arsenic resistance is stable and self-transmissible

We conducted mating experiments to determine if arsenic resistance was associated with a potentially self-transmissible genetic element as previously described for the *Y. enterocolitica* transposon *Tn2502* [21]. Where possible, recipient strains were chosen with a marker useful for counter selection (Amp^r^) against the donor. In other cases, a differential medium (Congo red agar) was used to screen mating mixes to phenotypically identify the recipient strain. The genetic background of the putative transconjugants was confirmed by PCR (*caf1*A, *lcr*V, *pla*) and/or biochemical analysis (glycerol fermentation).

When cultures of the arsenic resistant strain Java 9 were mixed with cultures of strain A4-3 (Amp^r^), ampicillin and arsenic resistant strains were obtained. 100% (25/25) of these isolates were Gly^+^ and *caf*1^+^ like the A4-3 strain. Similarly, when cultures of Java 9 (*pgm* ^+^ *lcr*V^+^ *pla* ^+^) were mixed with the *pgm* ^−^ *lcr*V^−^ *pla* ^−^ strain 1171-63, the resulting *pgm* ^−^ arsenic resistant strains had the same genetic profile as 1171-63. These transconjugants were capable, in turn, of transmitting arsenic resistant to recipient strains. Transconjugants exhibited arsenic resistance at a level similar to that of strain Java 9. Filter-sterilized whole DNA preparations or culture supernatants from Java 9 did not produce any arsenic-resistant strains when added to recipient cells. Plasmid profiles of transconjugants demonstrated that a ∼40 kb plasmid was present in the arsenic resistant strain derived from 1171-63 that was absent in the parental arsenic sensitive strain 1171-63. This plasmid co-migrated with the so-called “cryptic” plasmid of the Java 9 strain. Thus, it seemed likely that self-transmissible resistance to arsenic is mediated by this plasmid. Java 9 retained arsenic resistance after growth in the presence of the intercalating agent acridine orange and after growth at high temperatures, standard procedures to cure plasmids. Transconjugants were resistant to >100 mM arsenate and 5 mM arsenite.

### Plasmid inventory of Java 9

**Figure 1:**
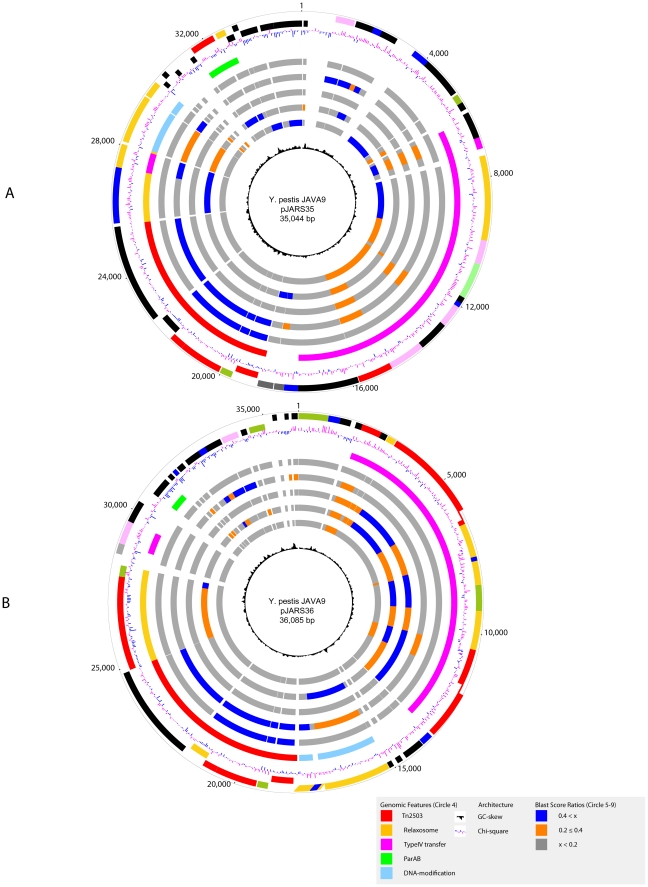
Conjugal transfer plasmids. Circles from outer to inner circles for (**A**) pJARS35 and (**B**) pJARS36: (circles 1 and 2) predicted coding sequences on the plus (1) and minus strands (2), colored according to the respective MANATEE role IDs. (circle 3) GC-skew. (circle 4) Plasmid features. *Tn2503* insertion (red). (circles 5 to 9) Comparative plasmid analysis to *Y. enterocolitica* pYVe227-*Tn2502* (circle 5), pJARS36 (circle 6), *A. culicicola* pAC3249-TypeIV (circle 7) and the *E. coli* plasmids R721 (circle 8) and R6K (circle 9). Chi-square (circle 10).

To determine the molecular nature of the arsenic resistance phenotype in strain Java 9, all indigenous plasmids were sequenced. Genomic analysis revealed that *Y. pestis* Java 9 is distinguished from all other currently analyzed *Y. pestis* strains by its unique plasmid inventory and composition (**Fig. 1**, **Fig. S1**). This strain carries two novel previously unknown plasmids, termed pJARS35 and pJARS36 (**Fig. 1**) that show no significant similarity with known *Yersinia* plasmid sequences and architectures [4], [18], [31], [32], [33], [34], [35] (**Fig. 1**). The mobilome of *Y. pestis* Java 9 features the integration of a transposon that is responsible for the arsenic resistance phenotype (**Fig. S2**). Interestingly, this 6,769 bp transposon is an integral part of all four indigenous plasmid molecules (pCD, pPCP, pJARS35 and pJARS36), and its insertion leads to length polymorphisms when compared to the typical *Y. pestis* virulence plasmids (**Fig. 1**, **Fig. S1**). The presence of arsenic resistance genes on each of the plasmids within this strain likely contributes to the stability of the arsenic resistance phenotype in our plasmid-curing attempts. The transposon displays homology to *Tn2502*, a transposon previously found in certain serotypes of *Y. enterocolitica* [21], and was thus termed *Tn2503*. The plasmid features are summarized in **Table 1** and compared to the *Y. enterocolitica* plasmid pYVe227-Ars(+). In addition to pJARS 35 and pJARS 36, strain Java 9 harbors the typical *Y. pestis* plasmids pPCP (plasminogen activator) and pYV/pCD1 (*Yersinia* virulence/calcium dependence) (**Fig. S1**). However, it lacks the species-specific plasmid pMT (pFra) that encodes the so-called murine toxin (phospholipase D), genes associated with biofilm formation, as well as the F1 capsule [36]. The pJARS plasmids are stable in strain Java 9 under standard laboratory cultivation. We attempted to cure Java 9 of its plasmid by repeated incubation at high growth temperature (39°C) or in the presence of chemical agents, such as ethidium bromide (EtBr) or novobiocin, and no curing was observed (data not shown).

**Table 1:** Plasmid content of *Y. pestis* Java 9 and comparison to the *Y. enterocolitica* W22703 plasmid pYVe227-*Tn2502*.

Strain		*Yersinia pestis*Java 9				*Yersinia* enterocolitica W22703
Biovar/Serotype		Orientalis				1B
Geographic Origin		Gempolan, Java				Belgium
Source		Dead Javanese Rat (*Rattus rattus diardi*)				Human
Year		1957				1972
Plasmids		**pPCP**	**pCD**	**pJARS36**	**pJARS35**	**pYVe227**
Genome Size	[bp]	16.033	77.077	36.085	35.044	69.673
GC-content	[%]	47.7	45.4	46.7	43	44.2
**Predicted Coding Sequences**						
Predicted Number		26	113	51	47	112
Coding Area	[%]	83.1	82.6	90.5	86	81.3
Average Length	[bp]	512	563	640	640	506
**Arsenic Resistance**						
Transposon		*Tn2503*				*Tn2502*
Genome Size	[bp]	6.769				4.623
GC-content	[%]	51				52
Mobility locus		*tnpAR*				*tnpAR, degenerate*
Resistance locus		*arsHRBC*				*arsHRBC*

### Novel plasmid architectures and coding capabilities

The pJARS5 and pJARS36 plasmids each carry phylogenetically unrelated origins of replication (*ori*) with homologs to corresponding loci in other enterics. The pJARS35 harbors the plasmid initiation replication protein (PIR) and a genetic organization similar to that of *E. coli* IncX-type R6K plasmid [37], [38] (Fig. 1A). The pJARS36 *ori* is characterized by a DNAJ-like chaperone and a TrfA-family transreplication factor, such as that found in the small cryptic *Y. pestis* plasmid pYC and, more recently, in *Photorhabdus asymbiotica* [39], [40] (Fig. 1B).

**Figure 2:**
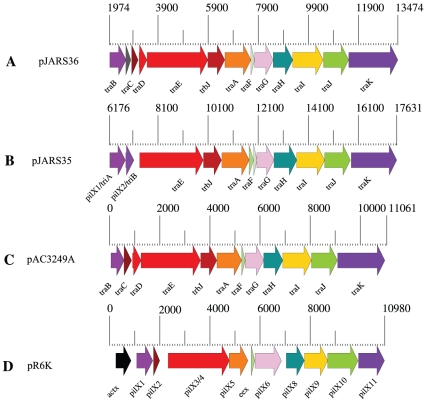
Genomic architecture of the conjugal transfer system. The conjugal transfer system on plasmids pJARS36 (**A**) and pJARS36 (**B**) share high homology and synteny with corresponding plasmid-borne loci in the insect inhabitant *A. culicicola* pAC3249A (**C**) and *E. coli* R6K (**D**). The scale in base-pairs indicates the respective genomic location of the plasmid–borne type IV systems. Genes shared between these loci are highlighted with similar colors.

These novel plasmids carry type IV conjugal transfer systems composed of 13 genes (**Fig. 1**, **Fig. 2**). As evidenced by the genomic analysis of these loci, the operons show high protein conservation and syntenic organization to corresponding loci of the potentially niche-sharing insect inhabitant *Aeromonas culicicola* [41] and *E. coli* pR6K. We note that *A. culicicola* was initially isolated from blood-feeding mosquitoes [41]. This finding supports the notion that the midgut of insects presents a potential suitable environment for genetic exchange and lateral transfer driving *Y. pestis* genome evolution [42].

### Transposon architecture and coding capabilities

**Figure 3:**
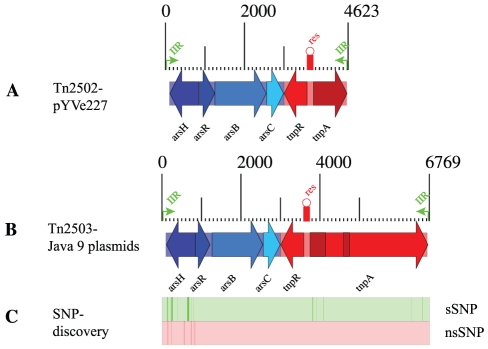
Transposon architecture and coding capabilities. The *Y. enterocolitica* transposon pYVe227-*Tn2502* (**A**) shows homology to *Tn2503* carried on all four indigenous Java 9 plasmids (**B**). Comparison of the *Yersinia*-derived arsenic resistance transposons reveals a defect mobility loci in *Tn2502*. SNP discovery (**C**) identified the *arsH* gene as mutational hot spot. Genes shared between these loci are highlighted with similar colors., arsenic resistance and mobility loci are marked in blue and red. IIR, flanking imperfect inverted repeats (IIR). res, resolution site.

Borders of the *Tn2503* are defined by 53-bp imperfect inverted repeats (IIR) (**Fig. S2**). Mobility and resistance loci are an integral part of the *Tn2503* transposon architecture, encoding a resolvase-transposase *tnpRA* and the resistance operon *arsHRBC* (**Fig. 3**, **Fig. S2**). *Tn2503* encodes a typical *arsHRBC* four-gene system that mediates arsenic resistance and is found in many prokaryotic genomes [19], [43] (**Fig. 3**). The system is comprised of the arsenite transporter ArsB, the arsenate reductase ArsC that converts arsenate to arsenite, the regulator ArsR, and the protein ArsH of unknown physiological function [44]. We identified an internal resolution (*res*) site that is located within the inversely oriented *tnpRA* mobility genes (**Fig. 3**). This region is characterized by three IIR of different length (**Fig. S2**). Transposon-mediated arsenic resistance has been previously reported in *Leptospirillum ferriphilum* and *Acidithiobacillus caldus* [45], [46] isolated from a commercial biooxidation tank in South Africa. However, these transposons are phylogenetically unrelated to *Tn2503*. Genomic analysis revealed a phylogenetic relationship to transposon *Tn2502* carried on the *Y. enterocolitica* strain W22703 virulence plasmids pYVe 439-80 and pYVe227 [21], and to the mobility locus of the *E. coli Tn2501* transposon [24], [47], [48] (**Fig. 3**).

### Transposon polymorphisms

Comparative analysis detected fine polymorphisms between the *Yersinia*-carried transposons *Tn2503* and *Tn2502*, which share an overall nucleotide identity of 93.25%. Two intragenic deletions (*tnpA* coordinates: 1–130, 840–994) in *Tn2502* result in a defective mobility locus (710 aa). The truncated *Tn2502* transposase pseudogene is nonfunctional; however, transposition has been restored in-trans by *Tn2501* in *Y. enterolitica* [21]. To study fine polymorphisms that may affect the arsenic sensitivity, we deployed a bioinformatics pipeline for mutation discovery and compared both transposon sequences [4] (**Fig. 3C**). This approach detected 30 synonymous (sSNPs) and seven non-synonymous SNPs (nsSNP) (**Table S1**).

Interestingly, the detected SNPs are non-randomly distributed (**Fig. 3C**). When comparing transposons *Tn2503* to *Tn2502* the *arsH* gene is most polymorphic among the arsenic resistance mediating genes and carries all nsSNPs as well as the majority of sSNPs. In contrast the remainder of the *arsRBC* operon is highly conserved (**Table S1**, **Fig. 3**). We note here that this protein is specifically required for arsenic resistance in *Y. enterocolitica* [21], which also might hold true in the genomic context of Java 9. While its physiological function remains unclear, the observed polymorphisms may impact the strains' overall arsenic resistance capabilities. However, the physiological effects of the detected polymorphisms in the *arsH* gene are unclear from the current literature and its biological role in mediating arsenic resistance needs further experimental evaluation. This finding may argue for a high selective pressure on the remainder of the arsenic resistance genes (*arsRBC)*.

### Dosage effects and arsenic resistance

The resistance phenotype is genetically linked to the presence and prevalence of the *Tn2503* transposon. The complete *Tn2503* transposon sequence and its annotation is visualized in **Fig. S2**. We speculate that gene dosage effects of *Tn2503* might be associated with the pronounced As resistance phenotype that we observed in strain Java 9. Strain Java 9 carries four transposon insertion in contrast to the single *Tn2502* copy found on the *Y. enterocolitica* pYV plasmid showing less arsenic resistance [21]. The transposon copies on each of the four indigenous plasmids (**Fig. 1**, **Fig. S1**) are increased *in vivo* considering the plasmid ratios in the sequenced DNA preparation, which we estimated in a ratio of 1∶2∶3∶2 for pCD, pPCP, pJARS36 and pJARS36 based on their sequence read coverage in the shotgun genome sequence.

### Specificity of *Tn2503* insertion

**Figure 4:**
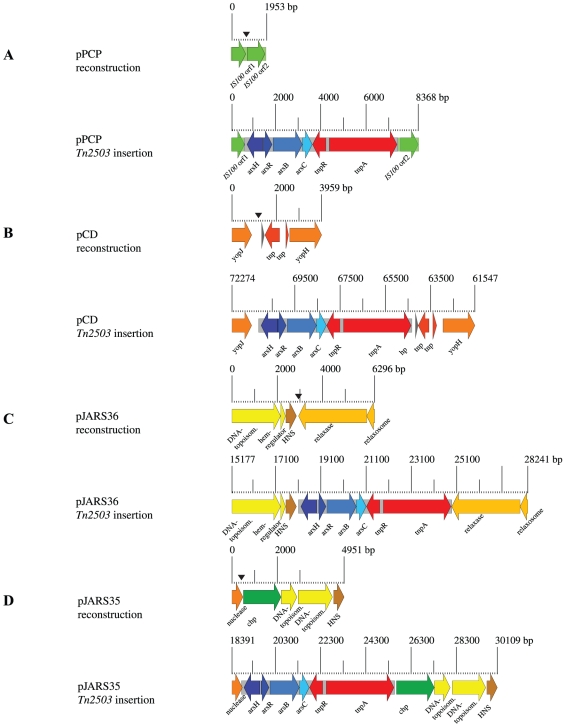
Target sites of transposon *Tn2503*. After excision of *Tn2503* at the target sites (black wedge), the plasmid states prior transposition for all four indigenous Java 9 plasmids were reconstructed to delineate the effects on neighboring genes. The transposon is found intergenic on pPCP disrupting the *IS100* sequence (**A**) and the conjugal transfer plasmids pJARS35 (**C**) and pJARS36 (**D**), while on pCD (**B**) insertion leads to truncated fertility inhibition protein FinO. The scale in base pairs indicates the respective genomic location of the *Tn2503* insertion loci. Genes shared between these loci are highlighted with similar colors.

Genomic analysis of the transposon insertion sites identified four target regions that appear to have been acquired by lateral acquisition (**Fig. 4**). To investigate the effects caused by the *Tn2503* insertion, we excised the transposons and reconstructed the plasmid states before integration *in silico* (**Fig. 4**). Their characteristic genomic features are a deviating GC-content when compared to the plasmids overall GC-content (**Table 1**) due to co-localization with AT-rich mobility loci, such as an *IS100* element (YPJ_pPCP1, YPJ_pPCP8) on pPCP, or two transposase remnants on pCD (YPJ_pCD105, YPJ_pCD105) (**Fig. 4**, **Fig. 1**, **Fig. S1**). In three instances, the transposon was found to be intergenic, while on pJARS35 the transposon insertion led to a C terminal truncation of 9 aa of a nuclease (YPJ_pJARS3526, 150 aa) when compared to the reconstructed locus prior insertion (159 aa) (**Fig. 4**, **Fig. 1B**). Of note, in pJARS36 and pJARS35, this transposon is integrated in proximity to the terminus of replication. However, intragenic insertions of the AT-rich *Tn2503* transposon may impact regulation and alter expression levels of neighboring genes.

## Discussion

Owing to the serious impact of *Y. pestis* on human health, additional sequence information is important for examining genomic plasticity at the level of individual polymorphisms. *Y. pestis* can become highly resistant to clinically useful antibiotics and other toxic compounds by the acquisition of genetic material originating from different phylogenetic sources [34], [40], [49]. Here we describe novel genetic traits in the mobilome of strain Java 9 that are associated with its high arsenic-resistance phenotype. We identified a promiscuous class II transposon as the resistance locus, and discovered two novel self-transmissible plasmids indicative for an open panmobilome of *Y. pestis* previously unknown to be part of the *Y. pestis* genome pool [4].

### Plasmid repertoire

Its unique mobilome provides further evidence of a dynamic plasmid repertoire in *Y. pestis* that is not restricted to the classical virulence plasmids [49]. This plasmid set introduces more than 100 genes that enable niche-adaptation and increase bacterial fitness and supports the notion that the plasmid inventory is a major driver of genome evolution in *Y. pestis* [50]. This finding is further supported by reports of atypical plasmids carried in distinct *Y. pestis* strains [4], [49], [50], [51]. We observed a likely genetic dissemination of genetic material among the Java 9 pJARS plasmids and other niche-sharing bacterial pathogens, either due to common ancestry or lateral exchange, as evidenced by its shared replication and transfer loci [34], [40], [49], [52], [53], [54]. The detected shared plasmid loci between Java 9 and *A. culicicola* support the notion that the gut of blood-feeding insects could act as a potential site of genetic exchange driving *Y. pestis* microevolution [41], [42]. The type IV loci may enable self-transmission of these plasmids and thus promote the spread of arsenic resistance (and associated genes) within and between species. Plasmid analysis provides evidence for a secondary lateral acquisition in the evolutionary history of *Y. pestis* since divergence from *Y. pseudotuberculosis*. In accordance, we noted the absence of IS elements in the novel conjugal transfer plasmids that underwent a massive expansion during *Y. pestis* speciation and are typically found on all *Y. pestis* DNA molecules.

### Plasmid pMT-deficiency in *Y. pestis*

The ability of this pMT-deficient strain to cause lethal plague in the primate model has major public health implications [13]. This strain would evade standard F1-based immunodiagnostic assays, such as previously reported by our group for the capsular antigen-deficient Pestoides (0.PE3 branch) strain Angola [4], [5]. In cases, when other DNA-based detection assays are not readily available this F1 negative strain characteristic could potentially delay crucial antibiotic treatment. Besides Java 9, other pMT-deficient and thus non-encapsulated *Y. pestis* strains have been reported from natural sources [4], [55], [56]. The pMT-borne capsule is thought to be involved in *Y. pestis* pathogenesis, in particular by inhibiting phagocytosis [57], [58]. This antigen is used as valuable biomarker in diagnostic assays. Because the pMT plasmid is highly conserved among *Y. pestis* species, it is possible that the capsule could conceivably be an important virulence factor in certain rodent hosts in nature. However, it is not a prerequisite for full virulence, as evidenced in our previous analyses of the F1-deficient *Y. pestis* strains Angola, C12, and Java 9 in rodent and non-human primate animal models [4], [26], [57], [59], [60]. Despite the lack of the pMT plasmid, this particular strain is capable of forming biofilms in the *Caenorhabditis elegans* nematode model, which is of major importance for the *Y. pestis* life cycle during flea borne transmission [4], [61], [62], [63].

### Transposon Tn2503

We noted that all four *Tn2503* transposon sequences integrated on each of the plasmids are genetically identical, which might be caused by selective pressure, gene conversion, or recent acquisition. The phylogenetically related *E. coli* transposon *Tn2501* is activated in a temperature-dependent manner through site-specific recombination [64], [65], which might also hold true in the genetic background of strain Java 9. We detected *Tn2503* transposon insertions causing mutations, which is exemplified by the intergenic *Tn2503*-pCD insertion between the *Yersinia* outer membrane (Yop) proteins J (YPJ_pCD114, 288 aa) and H (YPJ_pCD104, 468 aa) (**Fig. 4**). These surface-exposed proteins are intimately associated with the bacteria-host interactions, and the discovered alterations in these regulatory regions may impact pathogenesis and antigenic capabilities [66], [67].

### Environmental exposure to arsenic compounds

This resistance transposon is part of a floating mobilome as evidenced by its presence in *Y. enterocolitica* and *Y. pestis* [21]. Transposon carriage enables these particular strains to tolerate increased levels of toxic arsenic compounds. Acquisition of the resistance transposon might have been triggered by the natural or man-made environmental exposure to arsenic compounds. It is interesting to note that arsenic-based therapeutics have been used in veterinary medicine to cure *Y. enterocolitica* infections. Swine represent a major host reservoir of *Y. enterocolitica*, and arsenic therapeutics were historically used against swine dysentery, caused by *Serpulina hyodysenteriae*, or to increase meat production [21]. In Indonesia, rodents impose a major threat to agricultural crop production. Rodenticides are often based on arsenic compounds-, and are widely used in pest control [68], [69], [70]. We speculate that the strain Java 9 may have been exposed to toxic arsenic levels in its rodent host reservoir in similarity to the *Y. enterocolitica*.

The recent emergence of previously unknown *Y. pestis* genotypes, some of which are associated with altered pathogenicity and niche-specific adaptations should be considered in the future plague surveillance, prophylaxis and treatment [4], [49], [51]. In contrast to the reductive evolution in *Y. pestis* since divergence from its progenitor *Y. pseudotuberculosis*, this study underlines the major impact of a dynamic mobilome and lateral acquisition of genetic material including complete plasmids in the genome evolution of the plague bacterium.

## Supporting Information

Figure S1 **Virulence plasmids.** (**A**) Plasminogen activator plasmid pPCP. Circles from outer to inner circle: (circles 1 and 2) predicted open reading frames encoded on the plus (circle 1) and minus strands (circle 2), colored according to the respective MANATEE role IDs (circle 3) GC-skew. (circle 4) *Tn2503* insertion (red) disrupting *IS100* (green). (circles 5 to 9) Comparative plasmid analysis to *Y. pestis* strains CO92 (circle 5), KIM (circle 6), 91001 (circle 7), Antiqua (circle 8), Nepal516 (circle 9). Chi-square (circle 10). **(B). Low-calcium response plasmid pCD.** Circles from outer to inner circle: (circles 1 and 2) predicted open reading frames encoded on the plus (circle 1) and minus strands (circle 2), colored according to the respective MANATEE role IDs (circle 3) *Tn2503* insertion. (circles 4 to 10) Comparative plasmid analysis to *Y. pestis* strains CO92 (circle 4), KIM (circle 5), 91001 (circle 6), Antiqua (circle 7), Pestoides F (circle 8) and to *Y. pseudotuberculosis* pYV32593 (circle 9) and *Y. enterocolitica* pYVe227 (circle 10). Chi-square (circle 11).
(EPS)

Figure S2 **Transposon architecture.** The 6.769 bp transposon *Tn2503* is flanked by 53 bp imperfect inverted repeats (gray). Predicted genes are colored in green and code the arsenic resistance *arsHRBC* and mobility loci (*tnpRA*). The internal resolution site (res) is located between the divergently orientated transposases. It consists of three regions of imperfect dyad symmetry (purple).
(EPS)

Table S1 **List of SNPs.**
(XLSX)

Table S2 ***Y. pestis*** **strains used in this study draft.**
(DOC)

Table S3 **Primers used in this study.**
(XLS)
