# Supplementary material for: Novel Plasmids and Resistance Phenotypes in Yersinia pestis: Unique Plasmid Inventory of Strain Java 9 Mediates High Levels of Arsenic Resistance
Source: PLoS One. 2012 Mar 30;7(3):e32911. doi: 10.1371/journal.pone.0032911 (PMC3316555; doi:10.1371/journal.pone.0032911)
Supplement: Table S2 — Y. pestis strains used in this study draft. (DOC) [file pone.0032911.s004.doc]

**Table S2. *Y. pestis*** strains used in this study

| **Strain** | **Geographic origin** | **Year of isolation** | **Biovar** | **Resistance phenotype** | **Genotype/Phenotype metadata** | **Source** |
| --- | --- | --- | --- | --- | --- | --- |
| Java 9 | Java, Indonesia | 1957 | ORI | arsenic resistant | virulent, pMT (-) [F1(-)] | USAMRIID collection |
| CO92 | Colorado, USA | 1992 | ORI | arsenic sensitive | virulent | USAMRIID collection |
| La Paz | La Paz, South America | unknown | ORI | arsenic sensitive | virulent | USAMRIID collection |
| 1171 | Utah (fleas) | unknown | ORI | arsenic sensitive | attenuated (Pgm-, pMT-, pPCP-) | USAMRIID collection |
| KIM 10 | Kurdistan | unknown 1968 | MED | arsenic sensitive | attenuated (Pgm-) | USAMRIID collection |
| 195 P | India, Poona (human) | unknown | ORI | arsenic sensitive | virulent | USAMRIID collection |
| Antigua | Belgian Congo | unknown | ANT | arsenic sensitive | virulent | USAMRIID collection |
| Nairobi | Nairobi, Africa | unknown | ANT | arsenic sensitive | attenuated (Pgm-, pYV-, pMT-), | USAMRIID collection |
| Pestoides A | Former Soviet Union | <1984 | PESTOIDES | arsenic sensitive | virulent | USAMRIID collection |
| EV76 lot 4 | Madagascar, vaccine strain  Walter Reed | unknown | ORI | arsenic sensitive | live vaccine strain  (Pgm-) | USAMRIID collection |
| A1122 | California, USA  derivative of Yreka | unknown | ORI | arsenic sensitive | attenuated (Pgm- pYV-) | USAMRIID collection |
| Java 9 Lcr^-^ A | derivative | not applicable | ORI | arsenic resistant | Java 9 derivative cured of pCD | This study |
| Java 9 Lcr^-^ B | derivative | not applicable | ORI | arsenic resistant | Java 9 derivative cured of pCD | This study |
| 1171-63 | derivative | not applicable | ORI | arsenic sensitive | 1171-derivative cured of pCD | This study |
| A4-3 | derivative | not applicable | ANT | arsenic sensitive | Ampr-derivative of Antigua | Worsham, unpublished |
| 1171-63 pJARS1 | derivative | not applicable | ORI | arsenic resistant | transconjugant of Java 9 and 1171-63 | This study |
| A4-3 pJARS1 | derivative | not applicable | ANT | arsenic resistant | transconjugant of Java 9 and A4-3 | This study |
| A4-3 pJARS2 | derivative | not applicable | ANT | arsenic resistant | transconjugant of 1171-63 pJARS and A4-3 | This study |
